# Restructuring lung cancer care to accelerate diagnosis and treatment in patients vulnerable to healthcare disparities using an innovative care model

**DOI:** 10.1016/j.mex.2023.102338

**Published:** 2023-08-24

**Authors:** Jessica Copeland, Eliza Neal, Will Phillips, Sophie Hofferberth, Christopher Lathan, Jessica Donington, Yolonda Colson

**Affiliations:** a Division of Thoracic Surgery, Department of Surgery, Massachusetts General Hospital, Harvard Medical School, Boston, MA, United States; b Division of Thoracic Surgery, Department of Surgery, Brigham and Women's Hospital, Harvard Medical School, Boston, MA, United States; c Division of Medical Oncology, Department of Medicine, Dana-Farber Cancer Institute, Harvard Medical School, Boston, MA, United States; d Division of Thoracic Surgery, Department of Surgery, University of Chicago Pritzker School of Medicine, Chicago, IL, United States

**Keywords:** Non-small cell lung cancer, Integrative multidisciplinary care, Healthcare disparities, Social determinants of health, Novel healthcare delivery model, Lung Cancer Strategist Program (care model developed) based off of FastTrack (method used to develop the care model)

## Abstract

The diagnosis and treatment of lung cancer is challenged by complex diagnostic pathways and fragmented care that can lead to disparities for vulnerable patients. Our model involved a multi-institutional, multidisciplinary conference to address the complexity of lung cancer care in vulnerable patient populations. The conference was conducted using a process adapted from the problem-solving method entitled FastTrack, pioneered by General Electric. Conference attendees established critical social determinants of health specific to lung cancer and designed a practical care model to accelerate diagnosis and treatment in this population. The resulting care delivery model, the Lung Cancer Strategist Program (LCSP), was led by a lung cancer trained advanced practice provider (APP) to expedite diagnosis, surgical and oncologic consultation, and treatment of a suspicious lung nodule. We compared the timeliness of care, care efficiency, and oncologic outcomes in 100 LCSP patients and 100 routine referral patients at the same thoracic surgery clinic. Patient triage through our integrated care model transitioned initial referral evaluation to a lung cancer trained APP to coordinate multidisciplinary patient-centered care that was highly individualized and significantly reduced the time to diagnosis and treatment among vulnerable patients at high-risk for treatment delay due to healthcare disparities.

• To develop the Lung Cancer Strategist Program care model, we used a three-step (*Design, Meeting, and Culmination*), team-based, problem-solving process entitled FastTrack.
• An advantage of FastTrack is its ability to overcome barriers embedded within hierarchal and institutional social systems, empowering those closest to the relevant issue to propose and enact meaningful change.
• Under this framework, we engaged a diverse field of experts to assess systemic barriers in lung cancer care and design an innovative care pathway to improve the timeliness and efficiency of lung cancer care in patients at risk for healthcare disparities.

## Graphical abstract

**Figure:**
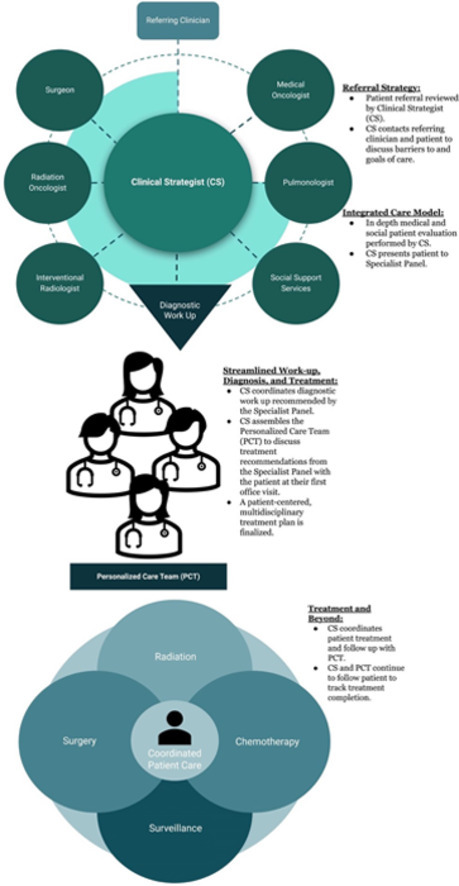

Specifications Table

**Table:** 

Subject Area:	Medicine and Dentistry
More specific subject area:	Healthcare Delivery
Method name:	Lung Cancer Strategist Program (care model developed) based off of FastTrack (method used to develop the care model)
Name and reference of original method:	*FastTrack (formerly Workout), pioneered by General Electric, was applied to facilitate problem-solving across a multidisciplinary panel of experts to result in the design and implementation of a novel healthcare delivery model.* **References and Applications of FastTrack:** Joseph, J. & Ocasio, W. Architecture, attention, and adaptation in the multi-business firm: General Electric from 1951 to 2001. Strategic Management Journal vol. 33 (2012). Henderson, K. M. & Evans, J. R. Successful implementation of Six Sigma: benchmarking General Electric Company. Benchmarking: An International Journal 7, (2000). Ngoie, O. M. General Electric case study - case study. DBA Program (2014). Clinton Health Access Initiative. Eliminating Mother-to-Child Transmission of HIV: CHAI's "across the cascade" approach. 2011; http://www.clintonhealthaccess.org/files/CHAI-eMTCT-fact-sheet-nov-2011.pdf. Schaninger Jr WS, Harris SG, Niebuhr RE. Adapting General Electric's Workout for Use in Other Organizations: A Template. Management 1999;2(1):99.
Resource availability:	Not applicable

## Introduction

Lung cancer is the leading cause of cancer-related death in the world killing three times more Americans than any other cancer [1,2]. The five-year survival rate for patients with lung cancer is approximately 15%, drastically lower than the 64% survival rate of colon, 89% of breast, and 99% of prostate cancers [2,3]. Although these differences in survival rates are daunting, survival nearly quadruples when lung cancer is diagnosed and treated at an early stage, with a 5-year survival rate of 60–70% [4].

Like many diseases, lung cancer survival is impacted by differences in accessibility and quality of care [5], [6], [7], [8], [9]. Data show that for vulnerable populations the incidence, prevalence and mortality rates for lung cancer are significantly higher compared to the general population [8,10,11]. Health disparities in lung cancer are multifactorial and affect all aspects of care, from screening and diagnosis, to treatment, survivorship, and end of life care. Social determinates of health that have emerged as key facilitators of health disparities include: cultural and biologic differences, systemic and structural impacts of race and class, inaccessibility to care and communication style [7,12,13]. Strikingly, these disparities still persist in equal access healthcare systems such as Medicare [14], [15], [16], [17], [18], [19].

The recent extension of lung cancer screening guidelines by the USPSTF is expected to more than double the number of Americans that qualify for lung cancer screening which will further compound the disparities and inefficiencies associated with lung cancer care [20]. Currently, 2 million Americans are diagnosed with a new pulmonary nodule annually [21], and an estimated 80,000 require surgical evaluation for malignant potential [22]. Although the inclusion of patients with lower smoking history significantly increases the numbers of minorities and women eligible for screening, the challenge for medical centers to provide high-quality, equitable lung cancer care to disadvantaged socioeconomic populations will be magnified by the increasing demand for limited oncology services and high costs associated with providing highly specialized and resource-intensive services.

Accordingly, it is crucial to implement novel care pathways that result in timely delivery of specialized thoracic oncologic care and are specifically constructed for populations that are vulnerable to having limited access to health care or encounter significant barriers to care. Novel pathways must work to streamline diagnostic and treatment pathways to prevent overloading of the system, provide diagnosis and treatment at the earliest stages of disease, and alleviate the impact social determinants of health have on lung cancer survival rates to assure equitable access to curative therapies. Patient navigation has emerged as a sustainable solution to minimize barriers in healthcare delivery and improve outcomes in vulnerable patients at high-risk for healthcare disparities. Such programs have proven helpful for vulnerable patients with breast, cervical, colorectal, and prostate cancer resulting in reduced patient anxiety, increased rates of cancer screening, and improved clinical outcomes through delivery of timely care [9,11,[23], [24], [25], [26], [27]]. However, patient navigation requires that the care pathways be relatively established and non-variable which is often not the case in lung cancer. The optimal clinical approach for a newly identified suspicious pulmonary nodule frequently involves complex decision making and diagnostic work-up that remains subject to provider interpretation, resulting in cancer care that is commonly delivered through multiple disorganized, clinical pathways.

To address these critical issues The Connors Center for Women's Health and Gender and the Division of Thoracic Surgery within Brigham and Women's Hospital and the Division of Medical Oncology at the Dana Farber Cancer Institute (DFCI) held a two-day conference centered on the design and implementation of a novel strategic care model to streamline the triage, diagnosis, and treatment of lung cancer in patients at high-risk for healthcare disparities. The objective was to listen to patients, caregivers, social organizations, healthcare providers, and government agencies to identify the key social determinants of health that impede care across the full spectrum of lung cancer and to implement a system that streamlines risk assessment, detection, diagnosis, and treatment [13,28]. Results from the conference yielded the design of the Lung Cancer Strategist Program (LCSP), a novel integrated care model that incorporated a single point of contact for expedited patient triage, risk assessment, and formulation of a multidisciplinary diagnostic and treatment plan. The efficacy of the LCSP to provide care to patients with non-small cell lung cancer (NSCLC) at high-risk for healthcare disparities was assessed in terms of timeliness of care delivery, care efficiency, and treatment adherence.

### Conference approach

The *Innovative Clinical Pathways in Lung Cancer Care for Vulnerable Populations Conference* was a two-day, multidisciplinary event involving 17 institutions and 63 stakeholders and leaders in the field of thoracic oncology funded by the Agency for Healthcare and Research Quality (AHRQ). The conference focused on the dissemination and implementation of evidence-based guidelines and care tools related to the full spectrum of NSCLC-care in high-risk populations. To develop the LCSP, conference organizers used a three-step (*Design, Meeting, and Culmination*), team-based, problem-solving process entitled “FastTrack” pioneered by General Electric [29], [30], [31]. The FastTrack process has been successfully validated in the public health arena in initiatives such as the Clinton Foundation Program to reduce mother-to-child-transmission rates of HIV [28]. A major advantage of FastTrack is the demonstrated ability to overcome barriers embedded within hierarchal and institutional social systems. FastTrack empowers those closest to the relevant issue to propose and enact meaningful change [29,30]. Under this framework, we engaged key stakeholders in the design and implementation of effective solutions to improve the accessibility of lung cancer care and patient outcomes.

## FastTrack problem solving approach & outcomes

### Design stage

Prior to the conference, a Steering Committee, consisting of a multidisciplinary panel of leaders in their respective fields, gathered to organize and construct a conference that would produce practical and actionable results. The Steering Committee consisted of a diverse group of 13 members including: thoracic surgeons, medical oncologists, pulmonologists, primary care physicians, geriatricians, patient advocates, healthcare business leaders, and policy experts. Ultimately, the diversity of the Steering Committee was considered the strength and backbone for the success of the conference in achieving its primary objective.

Once formed, the Steering Committee met monthly to formulate the most relevant issues for discussion at the conference, key attendees critical to the conference's success, and structure the conference to assure the creation of a care pathway that was widely applicable to different clinical settings and among different vulnerable patient populations. Additionally, each Steering Committee member identified: (1) conference goals and expectations, (2) perspectives on barriers to successful lung cancer care and treatment, (3) ideas on how to improve lung cancer care and treatment from their respective role in lung cancer care, (4) experiences providing lung cancer care and/or working with vulnerable populations, and (5) key stakeholders, including patients, to recruit to the invitation-only conference for the design of a novel care pathway.

**Fig. 1:**
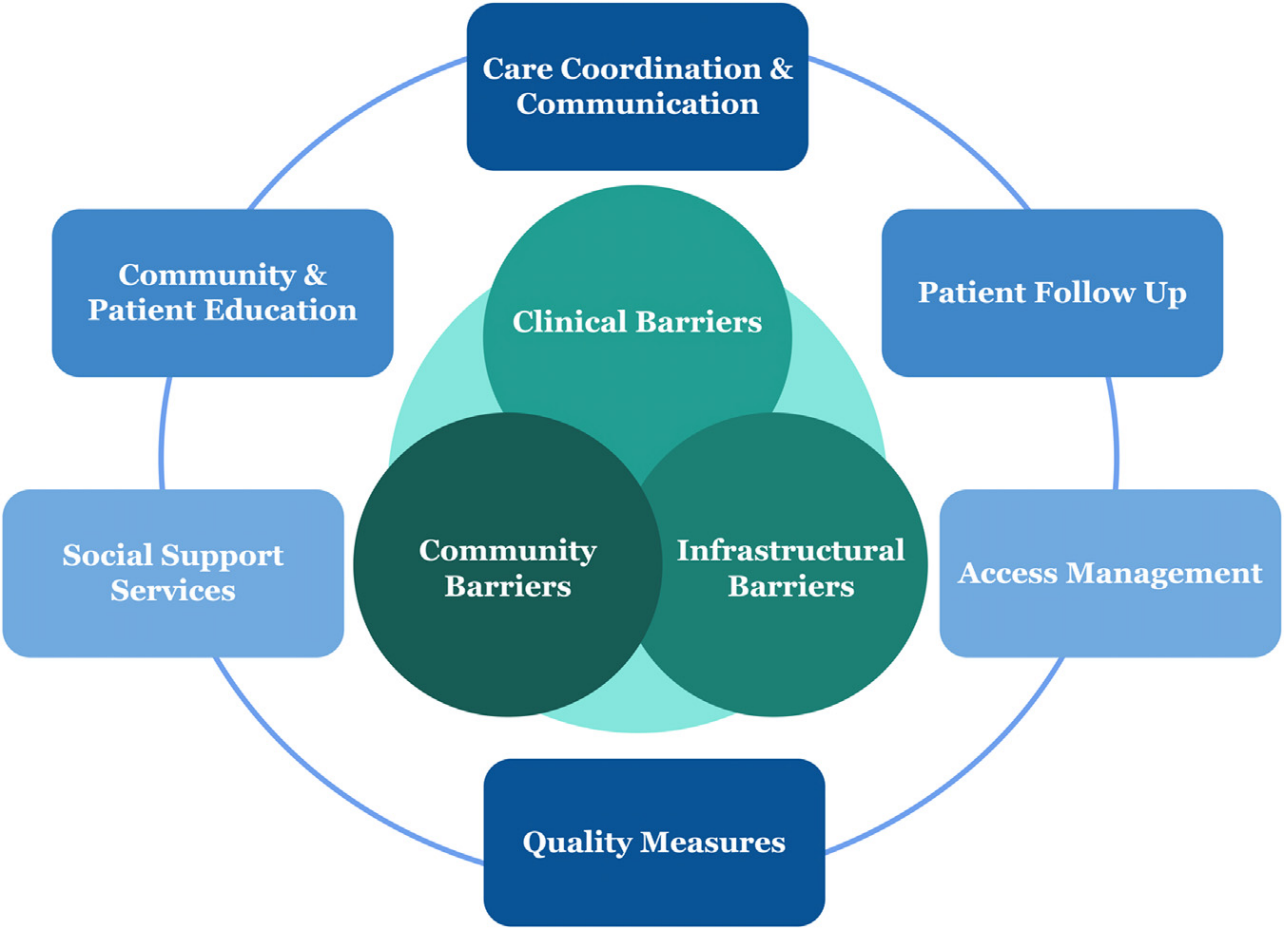
Integrating barriers (Venn Diagram) and solutions (Outer Circle) to common failure points across the spectrum of lung cancer care.

The Steering Committee's framework for the conference emphasized three pivotal points along the spectrum of lung cancer care where patient barriers were most likely to be magnified: 1) Clinical Barriers, 2) Community Barriers, and 3) Infrastructural Barriers. Within this framework, six areas of improvement were identified as essential components in establishing an effective and sustainable patient navigation pathway: 1) Care Coordination and Communication Solutions, 2) Patient Follow-Up Solutions, 3) Access Management Solutions, 4) Quality Measure Solutions, 5) Social Support and Service Solutions, and 6) Community and Patient Education Solutions (Fig. 1).

Based on this framework, the Steering Committee identified and recruited participants equipped to provide a broad range of perspectives and expertise in thoracic oncology and healthcare disparities. The final group of conference participants represented a cross-section of key stakeholders across a range of disciplines. In total, 63 participants attended the conference representing 17 independent healthcare institutions from 6 states and included physicians (surgeons, oncologists, pulmonologists, radiologists, internists), surgical and oncology nurses, clinical support staff, healthcare administrators, policymakers, patient advocates, as well as patients and their families.

### Meeting stage

**Fig. 2:**
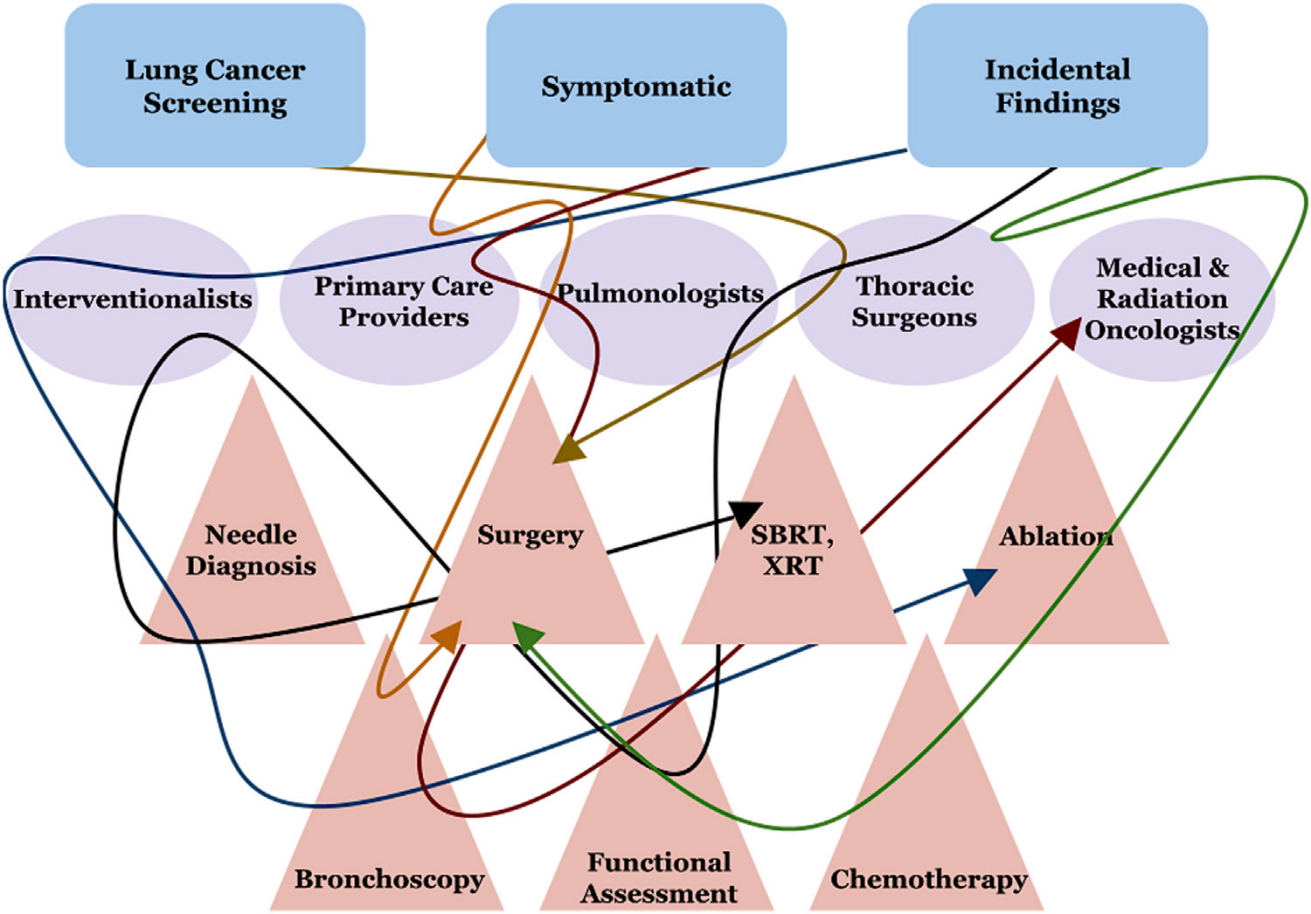
Current status of lung cancer care, lung cancer is one of the most complex clinical problems in the healthcare system. Patients can enter through multiple avenues and have multiple providers involved in the diagnosis, treatment, and maintenance of their disease.

The conference was divided into two sections: an idea exchange and data gathering workshop followed by a care model design and implementation workshop. To start the conference, attendees engaged in a key discussion on how lung cancer care is representative of current healthcare failures since it is difficult to navigate, manage, and organize timely care from the perspective of the patient, practitioner, and even the healthcare system itself. Patients with lung cancer enter the healthcare system through multiple avenues and with a wide range of clinical presentations, resulting in multiple providers with responsibility to coordinate care throughout diagnosis, treatment, and disease maintenance. By collectively analyzing multiple clinical scenarios and the care pathways it became evident that lung cancer is one of the most intricate yet fragmented clinical problems in the healthcare system (Fig. 2). Thus, providing a simplified and efficient lung cancer care model is an urgent healthcare imperative.

**Fig. 3:**
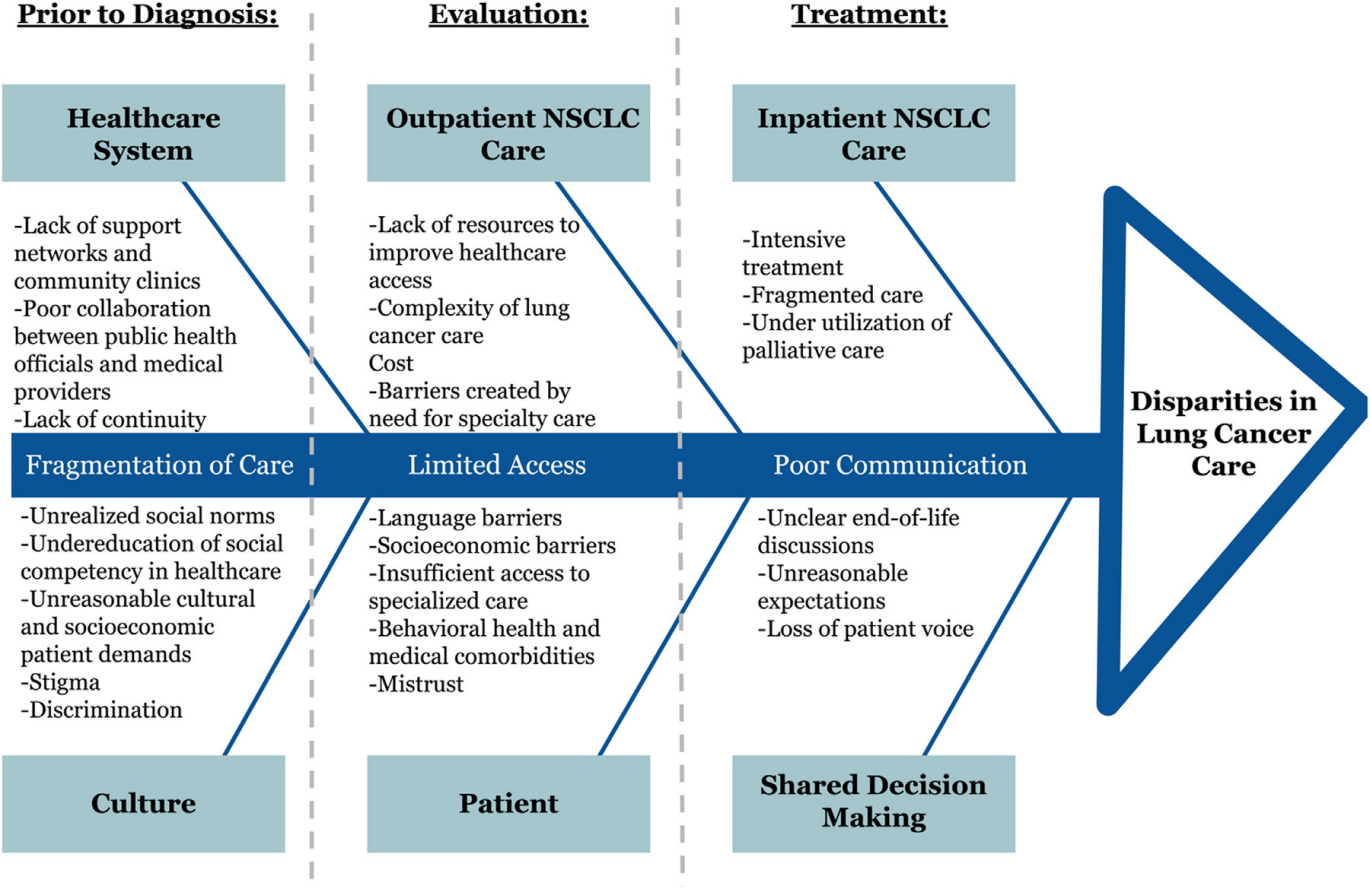
Fishbone diagram outlining key factors that increase healthcare disparities along the spectrum of lung cancer care.

During the idea exchange and data gathering workshop, participants were divided into teams of 8–10. Each team was facilitated by a Steering Committee member to identify health disparities and failures in care along the spectrum of lung cancer management. To maximize the identification of barriers, participants were organized into one of three barrier groups (Clinical, Infrastructural, or Community) by combining similar areas of expertise (Caregiver, Patient Advocate & Support, and Patient) to allow for a representative voice to emerge from each team (Table 1). This allowed us to identify the most significant barriers that were essential to address in the design of a novel patient care pathway. During this workshop common themes arose from all groups, including: fragmentation of care, poor communication, cultural differences, and accessibility to care. Additionally, each group highlighted the disease itself as being an inherent barrier- its current clinical complexity, the intensity of the treatments, and the urgency of intervention make it particularly difficult to manage, navigate, and cure. These barriers are graphically displayed in the fishbone diagram (Fig. 3), outlining the key clinical factors that increase the impact of health care disparity in the treatment of lung cancer.

**Table 1:** Conference teams divided into three groups to address specific type of barriers (Clinical, Infrastructural, Community) by combining similar areas of expertise (Caregiver, Patient Advocate & Support, and Patient).

CLINICAL TEAM:	INFRASTRUCTURAL TEAM:	COMMUNITY TEAM:
Primary Care Providers, Medical Oncologists, Radiation Oncologists, Pulmonologists, Thoracic Surgeons, Nursing Staff, Pain Management Specialists, Clinical Support Staff	Patient Care Coordinators, Payment and Financial Care Coordinators, Access Management Staff, Quality Improvement Staff, Health Information Technology Support	Patient Care Services, Patient Navigators, Smoking Cessation Counselors, Social Workers, Patient Advocates, Public Health Experts, Patients and Family Members

#### Insights from the clinical team

The clinical team observed that one of the central breakdowns in the spectrum of lung cancer care was the lack of patients as integral members of the care team. Although providers voiced openness and desire for shared decision-making, physicians found it difficult to explain the complexities and nuances of lung cancer without overwhelming patients. This issue was further exacerbated by patients interacting and receiving advice from multiple providers during separate sessions with each provider speaking about different treatment options in their respective field. Further, patients did not consistently perceive a welcomeness to participate in their care plan. Specifically, patients voiced that physicians did not always seem to know how to elicit or incorporate patient input. Therefore, the physician's difficulty in offering support and hope, being a team leader, and still being able to convey all the information needed to help the patient make an informed decision emerged as a prominent barrier. This issue is growing in importance as physicians experience greater time constraints and increased productivity targets.

#### Insights from the infrastructural team

The infrastructural team identified three major solutions and barriers consisting of: 1) Coordination of care to address the inherent complexity and fragmentation of lung cancer care; 2) Recognition that access and financial barriers are not isolated to a patient's insurance status and the need for upfront, individualized, social service assessment for potential barriers to care; and 3) Creation of a care delivery model where education and empowerment are built into the infrastructure. Additional factors they proposed to incorporate into the design of the care model were to increase the knowledge within the community about cancer risk, help patients make well-informed choices, and address emotional needs intrinsic to the fear and stigma that often accompany lung cancer. They also focused on the need for building a model that allowed patients to logistically and practically get the services and support needed throughout all phases of treatment (spanning home-, community-, hospital- and hospice-based care).

#### Insights from the community team

The community team highlighted barriers in three major areas: fragmentation of care, communication, and accessibility. The cohort observed fragmentation of care as a cause for unnecessary delays in diagnosis, the practice of multiple appointments to establish a care plan, and communication breakdown. They also voiced that while physicians provided resources for patient care, knowledge of resources within the community was not universal, and thus community support was also inconsistent and disparate. Community groups identified issues of fear, stigma, and health literacy as chief barriers in communication. Barriers created by access were expanded from transportation and income to include hardships imposed by loss of work due to multiple appointments, challenges of treatment, and complicated recoveries.

### Culmination stage

From these fundamental observations by each team, the Steering Committee developed a comprehensive but focused list of solutions to address each of the three key barriers (Clinical, Infrastructural, and Community), crucial in the implementation of a new clinical care model (Table 2). These solutions were constructed to also address another major conclusion of the conference, that patients at high-risk for healthcare disparities experience increased fragmentation of care, poor communication, and limited access. Solutions to successfully overcoming these barriers were concentrated on (1) coordinating care and improving communication across practitioners, (2) upfront social and community support assessment, and (3) establishing an integrative, multidisciplinary care team personalized to the patient.

**Table 2:** List of barriers and solutions to lung cancer care and treatment.


**Clinical Barriers:** Treatment: multiple appointments, procedures, and intensive and complex care. Coordination of care**:** lack of defined treatment process, designated care coordination, care coordinator, and communication.	**Care Coordination:** Patient is a pivotal team member. Designated care coordinator. Centralized care coordination. After visit summaries. Curbside consults for quick patient referrals. **Quality Measures:** Wait time intervals for consultative, diagnostic, and treatment services. Patient satisfaction and quality of life metrics. Institutional costs. Evaluation of current care models.
**Community Barriers:** Patient perception(s): mistrust, cultural and language barriers, fear, stigma, and denial. Patient experience(s): lack of sensitivity and communication skills among providers. Patient limitation(s)**:** transportation, child care, employment, income, health literacy, education, socioeconomic barriers, and life-altering situations.	**Social Support Services:** Health literacy assessment. Patient specific social support. Caregiver support. Psychosocial screening. Survivorship plans. **Community and Patient Education:** Patient education: prevention and importance of lung cancer screening. Provider education: knowledge and readily available information on community support services.
**Infrastructural Barriers:** Insurance: coverage and benefits. Smoking: screening and smoking cessation programs. Health care system: lack of connection between hospitals, providers, and community resources.	**Access Management:** Patient navigation system. Information technology resources (virtual visits) **Patient Follow-Up:** Provider scripts. Closed loop communication. Information technology resources (mobile applications, patient registries, scheduling and reminder systems, and computerized decision support).

#### Centralization of care and restructuring initial patient assessment

Given the complexity of lung cancer as a disease and of the fragmentation of care involved, the proposed solution of nearly all teams focused on a central figure whose role was to lead care coordination and establish a paradigm where the patient and primary care provider (PCP) are key members of the care team. Additionally, restructuring the traditional care pathway so that the initial consultation process for a suspicious pulmonary nodule was transitioned to the care coordinator was identified as fundamental in facilitating integrative care in terms of oncologic, surgical, and social service specialists and providing a multidisciplinary, patient-centered, treatment plan in a streamlined manner.

Developing a pathway centered on coordination of care highlighted the critical need for improved communication and support during key transitions in care (pre-diagnosis to diagnosis, surgical care to chemoradiation or vice versa, or from community to hospital to hospice care). Focusing on these transitions is crucial as this is where patient loss in the healthcare system is highest. Thus, it was crucial that our model identified a single contact person as the focal point for patients, providers, and the multidisciplinary care team. This would allow the care coordinator to serve as a conduit for the delivery of comprehensive clinical care in an individualized fashion.

By coordinating care through a central point, a multidisciplinary team of specialists could then establish an integrative, streamlined and clinically appropriate pathway for diagnosis and treatment that could in turn, be executed by the central coordinator. This paradigm was observed to have the potential to minimize patient time, effort, and risk while maximizing cost efficiency and diagnostic accuracy. Given the level of medical knowledge required for this responsibility and coordination, it was concluded that central coordination would require an advanced practice provider (APP) with expertise in lung cancer care to strategically execute the diagnostic care plan. Since a critical challenge in integrating a diagnostic and treatment pathway for lung cancer is founded on multiple pathways to diagnosis, centralization and early triage by thoracic trained specialists were deemed critical, as was the connection between patient and community provider in achieving improved navigation after a personalized plan was established. Accordingly, the role of referral pathways to specialized care and streamlined individualized diagnostic and care plans established and facilitated by an APP, were implemented as our primary intervention in our care model, the Lung Cancer Strategist Pathway (LCSP) and pilot study.

## LCSP care model implementation & validation

### LCSP approach

To address the inefficiencies and disparities specific to lung cancer care, we designed and implemented an integrated, patient centered, model of care, the Lung Cancer Strategist Program. LCSP was designed to minimize diagnostic redundancy, streamline management decisions for suspicious nodules, and expedite curative therapy for lung cancer patients at high-risk for treatment delay.

### Team and workflow

**Fig. 4:**
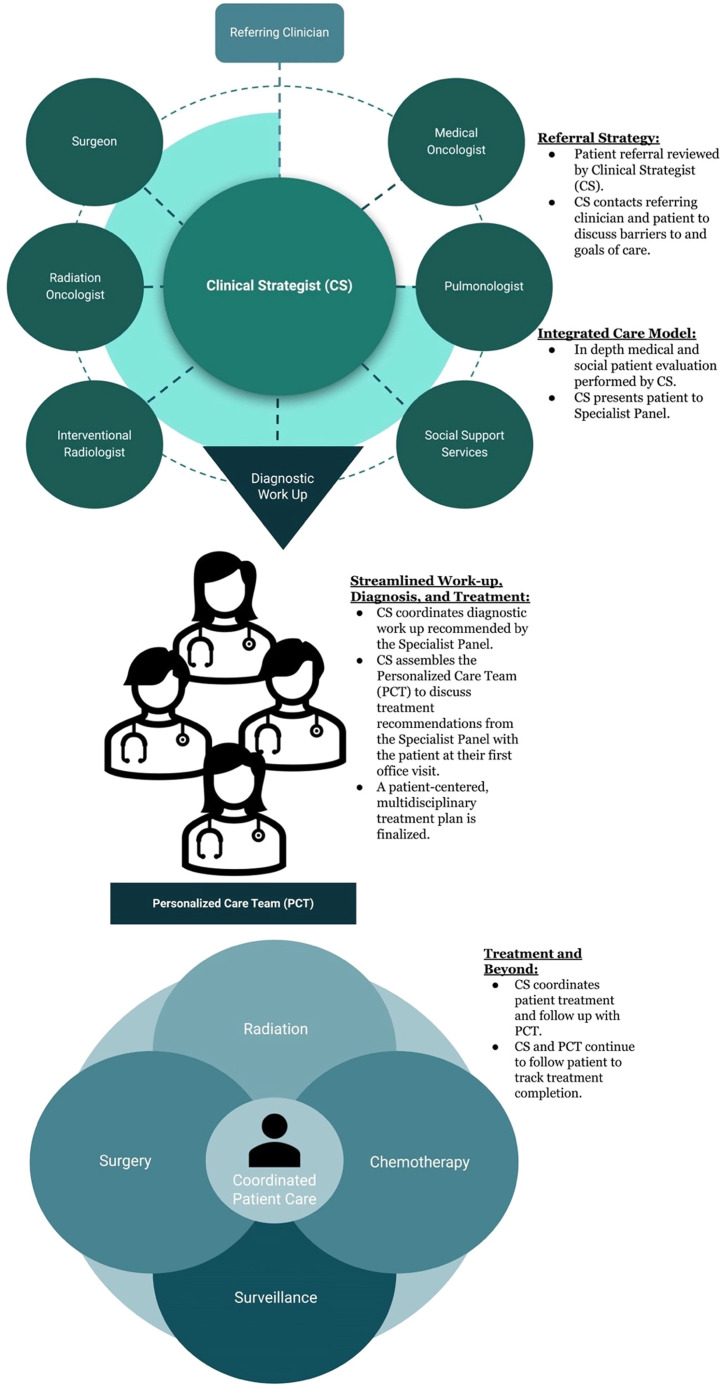
Overview and workflow of the lung cancer strategist program.

The LCSP approach was centered on coordinated evaluation of a suspicious lung lesion through a lung cancer-trained APP designated as the clinical-strategist (CS). In practice, the CS reviewed the patient's medical record following referral and presented the patient to a specialist panel to develop a customized evaluation strategy for each patient and ordered any necessary testing prior to the patient's first visit. The patient was evaluated at their first clinic visit by their Personalized Care Team (PCT), which was assembled by the CS and consisted of the appropriate oncologic, surgical, and social support specialists who reviewed results, discussed diagnosis, and implemented a multidisciplinary treatment plan (Fig. 4). This is distinctly different than coordination and navigation of the patient from provider to test and then to subsequent provider, rather this is the design of a strategic, efficient plan for the rapid diagnosis and treatment of lung cancer.

### Lung cancer strategist program an integrative care model strategy

The LCSP consisted of a multidisciplinary thoracic oncology team led by a lung cancer trained advanced practice provider and thoracic surgeon in consultation with oncology specialists. The rationale for pairing the APP with a thoracic surgeon rather than a medical or radiation oncologist is that surgical assessment is paramount in curative strategies and often in the diagnosis of lung cancer. Biopsy of a lesion when surgical resection is already warranted poses an unnecessary delay and risk. Therefore, the most expeditious means to curative resection for a highly suspicious nodule or a nodule which changes during surveillance is to have the surgeon involved in the initial assessment. This has the added benefit of allowing triage to radiation oncology (or other members in the PCT) for treatment or surveillance if the patient is not a surgical candidate, thus further decreasing delay for non-surgical treatments. We hypothesized that expedited diagnosis and treatment of a suspicious lung nodule could be achieved in vulnerable patients at high-risk for treatment delay when managed within the LCSP care model compared to patients who underwent routine referral at the same thoracic surgery clinic. Our model aimed to (1) restructure the initial consultation process after a suspicious pulmonary nodule was identified by transitioning the initial referral evaluation from specialist providers to APPs with lung cancer expertise, (2) incorporate oncologic and surgical specialists to create a multidisciplinary, patient-centered, treatment plan in a streamlined manner and (3) accelerate the timeliness of care and decrease procedures and care transitions in a vulnerable patient population at high-risk for experiencing treatment delay due to healthcare disparities amid fragmented, disorganized care.

**Fig. 5:**
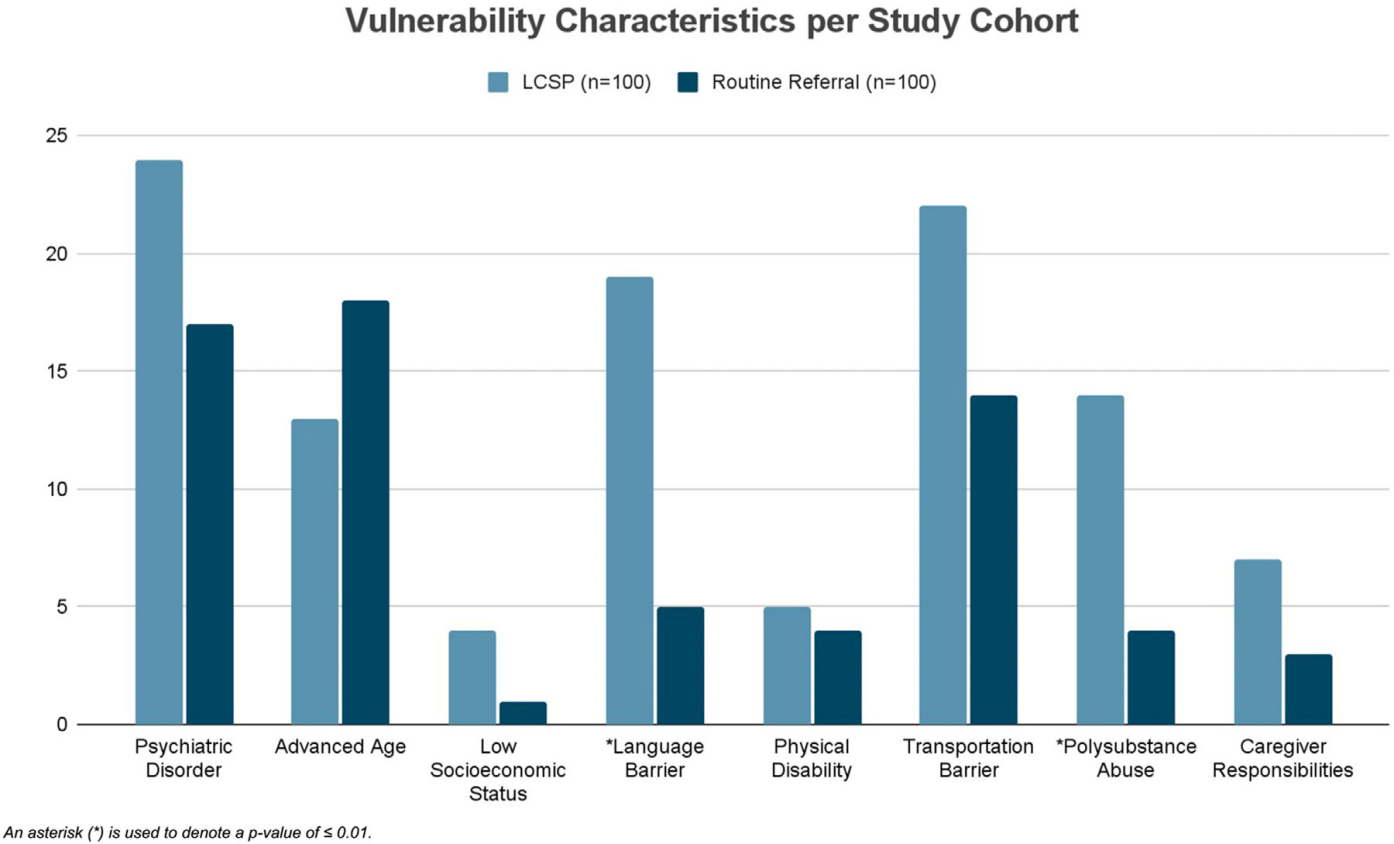
Patients were deemed high-risk for treatment delay based on vulnerability characteristics that are represented across the LCSP cohort and routine referral cohort.

Patients with a suspicious lung finding that were deemed high-risk for treatment delay, based on vulnerability characteristics, were prospectively accrued to the LCSP or to routine referral cohort. Lung nodules were risk stratified and identified as suspicious based on the Fleischner criteria. Vulnerability characteristics were defined based on patient factors identified in the literature associated with treatment delays including: psychiatric disorder, advanced age (>80 years old), low socioeconomic status, language barrier, physical disability, transportation limitation, polysubstance abuse, and caregiver responsibilities (Fig. 5) [32], [33], [34], [35], [36], [37], [38], [39]. History of polysubstance abuse and psychiatric disorders (schizophrenia, bipolar disorder, depressive disorder, and dementia) were documented according to the pre-existing diagnoses in the medical record. A language barrier was defined as persons with limited English proficiency, transportation barrier as an inaccessibility to a vehicle, physical disability as wheelchair-bound, blindness, deafness, and/or a history of falls, and low socioeconomic status was defined as those unemployed, uninsured, undomiciled, and/or with Medicaid health insurance.

Analysis of the first 100 patients managed in the LCSP versus the routine referral cohort within the same thoracic surgery clinic was performed. Primary outcome measures were timeliness of care delivery and care efficiency. Timeliness of care delivery was defined as the time interval from an initial suspicious lung finding to work-up, diagnosis, and definitive management plan (i.e., date of surveillance recommendation or initiation of treatment). Care efficiency was assessed by the number of hospital trips, clinicians seen, and diagnostic studies performed during work-up of the lung lesion. Secondary outcome measures were patient care adherence, stage at diagnosis, disease-free survival (DFS) and overall survival (OS). Pearson chi-square test was used to compare the percentage of patients in the LCSP and routine-referral groups. Timeliness of care and care efficiency metrics were compared using non-parametric Mann-Whitney *U* test. Independent binary proportions were compared using Fisher's exact test. Statistical analysis was performed with SPSS 23.0 (IBM Corporation, Armonk, NY). A P value < 0.05 was considered statistically significant.

### Model validation

In this report, we provide our methods for creating the LCSP, a multidisciplinary, patient-centered, integrated care model led by a lung cancer trained APP to streamline thoracic surgeon and oncologic specialist consultations and accelerate lung cancer diagnosis and curative therapy by optimizing access to medical care in patients identified as high-risk for treatment delay. In our prospective, non-randomized, single-center pilot study, the LCSP model significantly accelerated the time to diagnosis and treatment for patients with intrathoracic malignancies as well as the efficiency of care in patients who are at high-risk for healthcare disparities.

**Fig. 6:**
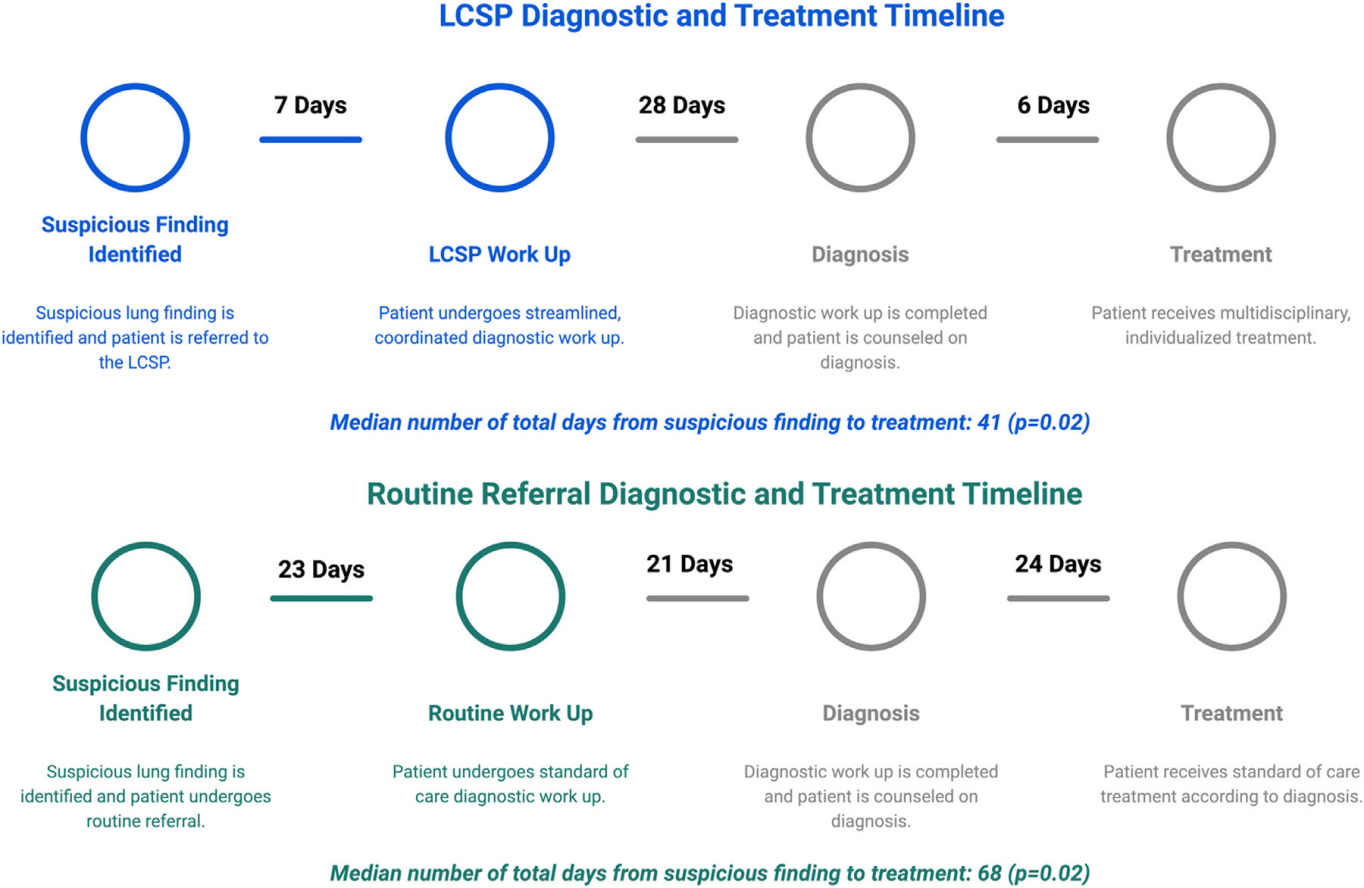
Diagnostic and treatment timeline for the LCSP cohort compared to the routine referral cohort.

The LCSP was superior in all measures of timeliness of care when compared to U.S. national standards and routine referral through the same thoracic surgery clinic. Overall, in the LCSP cohort we observed a median 7-day reduction from the time of patient referral to definitive management plan (12 vs. 19 days; *p* = 0.001) and 25-day reduction from the time of the initial suspicious finding to work-up (3 vs. 28 days; *p*<0.001) compared to the routine referral cohort. Further, treatment was provided at a median of 28 days earlier in the LCSP cohort diagnoses with malignancy compared to routine referral (40.5 vs. 68.5 days; *p* = 0.02). Care efficiency was also improved in the LCSP cohort by reducing healthcare redundancy in the form of hospital visits (4 vs. 6; *p* = 0.001) and diagnostic testing (4 vs. 5; *p* = 0.01) compared to the routine referral cohort, while the number of clinicians seen did not significantly vary (1.5 vs. 2; *p* = 0.08) (Fig. 6). Of the patients diagnosed and treated for NSCLC, there was no significant difference observed in clinical or pathologic stage at diagnosis or modality of treatment across cohorts. However, a higher rate of malignant diagnosis occurred in the routine referral cohort at 49% compared to the LCSP at 15% (*p*<0.001).

Improvements in timeliness of care delivery were attributed to the clinical strategist being able to provide expert lung cancer evaluation and act as a central point of contact for patients, referring providers, and oncology specialists. The rates of patient adherence and retention were similar at 83.3% and 82.9% for the LCSP and routine referral cohorts respectively, even with the LCSP's higher incidence of vulnerable features predisposing to care delays and non-adherence. However, the LCSP care adherence rate was superior to single institution studies reporting rates of 51–65% adherence and retention for patients enrolled in lung cancer screening programs with systematic policies in place to improve retention [26], [27].

However, we recognize this study has several important limitations. It was agreed that randomizing vulnerable patients to a pathway known to have treatment delays and fragmented care was undesirable and thus our study was a prospective, non-randomized, and non-blinded. This design impacted the referral patterns of each cohort. First, as referring providers became aware of the LCSP, patients at high-risk for healthcare disparities were more likely to be referred to the LCSP than the routine referral cohort. Despite, having a similar source population the routine referral cohort was still less heterogeneous in terms of patients at risk for healthcare disparities compared to the LCSP. Accordingly, the routine referral cohort potentially underestimates the impact that healthcare disparities have on the timeliness of routine care. Second, there was a disproportionate number of patients referred to the LCSP by emergency room physicians instead of primary care physicians. This potentially led to an overestimation of those requiring surveillance and underestimation of those needing treatment in the LCSP cohort. That is, patients referred by primary care physicians have often already undergone a surveillance period by the primary care physician and are referred once suspicious changes in surveillance are observed whereas, emergency room physicians are more likely to refer patients based on incidental findings which are more likely to be indolent in nature, primarily requiring surveillance.

Still, leveraging a broad range of expertise to systematically assess the social determinants and barriers to lung cancer care to create a novel healthcare pathway resulted in a significant improvement in care metrics for lung cancer patients at risk for healthcare disparities. Specifically, the LCSP significantly accelerated time to work-up, diagnosis, and treatment in a disease with a complex care pathway and in a patient population at high-risk for experiencing treatment delay.

## Conclusions

By restructuring the initial consultation processes and using an integrated care model, providers are able to facilitate shared decision making between the patient, referring physician, oncology specialists, thoracic surgeon, and social support services from the time of initial referral. This allows for the delivery of rapid, efficient, patient-centered, multidisciplinary clinical care in populations at high-risk for healthcare disparities.

## Ethics statement

The study was performed in accordance with ethical human research standards, informed consent was obtained by all study participants and was approved by the institutional review board at Brigham and Women's Hospital (IRB Number: 2019P000695).

## CRediT Author Statement

**Jessica Copeland**: Methodology, Model Design, Manuscript Preparation; **Eliza Neal:** Methodology, Reviewing and Editing; **Will Phillips**: Data Curation, Validity Testing; **Sophie Hofferberth**: Reviewing and Editing; **Christopher Lathan**: Model Design, Methodology; **Jessica Donington**: Model Design, Methodology; **Yolonda Colson**: Supervision, Editing.

## Declaration of Competing Interest

The authors declare that they have no known competing financial interests or personal relationships that could have appeared to influence the work reported in this paper.

## Data Availability

The authors do not have permission to share data.
